# The Insecticide Imidacloprid Decreases *Nannotrigona* Stingless Bee Survival and Food Consumption and Modulates the Expression of Detoxification and Immune-Related Genes

**DOI:** 10.3390/insects13110972

**Published:** 2022-10-22

**Authors:** Yahya Al Naggar, Humberto Estrella-Maldonado, Robert J. Paxton, Teresita Solís, J. Javier G. Quezada-Euán

**Affiliations:** 1 General Zoology, Institute for Biology, Martin Luther University Halle-Wittenberg, 06120 Halle, Germany; 2 Zoology Department, Faculty of Science, Tanta University, Tanta 31527, Egypt; 3 Departamento de Apicultura Tropical, Campus de Ciencias Biológicas y Agropecuarias, Universidad Autónoma de Yucatán, Mérida CP 97100, Mexico; 4 Instituto Nacional de Investigaciones Forestales, Agrícolas y Pecuarias (INIFAP), Campo Experimental Ixtacuaco, Km 4.5 Carretera Martínez de la Torre-Tlapacoyan, Tlapacoyan CP 93600, Mexico

**Keywords:** stingless bee, neonicotinoid, pollinator, decline, detoxification, pesticide

## Abstract

**Simple Summary:**

Stingless bees are the most diverse group of highly social bees, and they are ecologically and economically important species in the tropics and subtropics. Stingless bees provide important ecological services, such as the pollination of native plants and crops. However, agrochemical treatment is a common practice in the management of pest arthropods in many crops. Regrettably, little research has been conducted on the characterization of detoxification systems and immune responses in stingless bees, which is critical for understanding their responses to and defenses against a variety of environmental stresses, including agrochemicals. The purpose of this study was to investigate the effect of exposing the stingless bee *Nannotrigona perilampoides* to the commonly used neonicotinoid, imidacloprid.

**Abstract:**

Stingless bees are ecologically and economically important species in the tropics and subtropics, but there has been little research on the characterization of detoxification systems and immune responses within them. This is critical for understanding their responses to, and defenses against, a variety of environmental stresses, including agrochemicals. Therefore, we studied the detoxification and immune responses of a stingless bee, *Nanotrigona perilampoides*, which is an important stingless bee that is widely distributed throughout Mexico, including urban areas, and has the potential to be used in commercial pollination. We first determined the LC_50_ of the neonicotinoid insecticide imidacloprid for foragers of *N. perilampoides*, then chronically exposed bees for 10 days to imidacloprid at two field-realistic concentrations, LC_10_ (0.45 ng/µL) or LC_20_ (0.74 ng/µL), which are respectively 2.7 and 1.3-fold lower than the residues of imidacloprid that have been found in honey (6 ng/g) in central Mexico. We found that exposing *N. perilampoides* stingless bees to imidacloprid at these concentrations markedly reduced bee survival and food consumption, revealing the great sensitivity of this stingless bee to the insecticide in comparison to honey bees. The expression of detoxification (GSTD1) and immune-related genes (abaecin, defensin1, and hymenopteacin) in *N. perilampoides* also changed over time in response to imidacloprid. Gene expression was always lower in bees after 8 days of exposure to imidacloprid (LC_10_ or LC_20_) than it was after 4 days. Our results demonstrate that *N. perilampoides* stingless bees are extremely sensitive to imidacloprid, even at low concentrations, and provide greater insight into how stingless bees respond to pesticide toxicity. This is the first study of its kind to look at detoxification systems and immune responses in Mexican stingless bees, an ecologically and economically important taxon.

## 1. Introduction

Bees, both wild and managed, play an important role in pollinating a wide variety of agricultural and wild plants [1,2,3]. The honey bee (*Apis mellifera* L.) is the most widely used pollinator in commercial crops around the world, and it is regularly used as a model organism for nontarget toxicity studies [4,5,6,7]. However, major overwinter losses of honey bee colonies have been seen in different parts of the world since 2006, with a variety of biotic and abiotic stressors being cited as causes [8,9,10,11,12,13]. As a result, the employment of different bees, such as stingless bees, has been suggested for the tropics and subtropics, as these bees also pollinate agricultural crops [2,14,15,16].

The Meliponini, or stingless bees, are the most diverse eusocial taxon among the 20,000 bee species [17,18,19]. In terms of species, they outnumber honey bees by a factor of 50 (Apidae, Apini: 11 species [20]), and account for over twice the number of known bumble bee species (Apidae, Bombini: approximately 250 species [20]). Stingless bees provide important ecological services, such as the pollination of native plants and crops; many of the tropic’s most species-diverse plant families, including Fabaceae, Asteraceae, Rubiaceae, Poaceae, Euphorbiaceae, Myrtaceae, Malvaceae, Arecaceae, Solanaceae, and Anacardiaceae, have been reported to attract stingless bees to their flowers [16,21]. However, agrochemical treatment is a frequent practice in the management of pest arthropods in many crops of these plant families [22]. In addition, urban areas can be home to several species of stingless bee [23] and, as a result, exposure to pesticides for the control of insect vectors of human pathogens can be frequent in these environments [24].

Neonicotinoids belong to an insecticide class that has gained popularity due to claims that they are less toxic to humans and other animals. Insects, on the other hand, are very vulnerable to them since their neural acetylcholine receptors have a high affinity for neonicotinoids [25,26]. Exposure to neonicotinoids causes hyperexcitement and rapid mortality in insects by blocking acetylcholine receptors in their neurons [27,28,29]. In Mexico, the use of neonicotinoids started when imidacloprid was approved for use in 1993. Subsequently, in 2004, another neonicotinoid (thiamethoxam) was also endorsed in the country. Both neonicotinoids are mainly used on Solanaceae (potato, tomato, and peppers) for the control of whitefly (*Bemisia tabaci*) populations, a vector of viral diseases that impose considerable economic losses [30].

The systemic mode of action of neonicotinoids on pollinators is a key risk. They are absorbed by the roots of the plant and can become present in the nectar and pollen [31]. They can persist in the environment long after the initial application, resulting in chronic exposure to non-target insects [32], including bees that consume nectar and pollen as their principal sources of nutrition. Laboratory tests have shown that neonicotinoids are among the most toxic compounds to stingless bees [33,34,35,36]. Imidacloprid, in particular, is a well-studied neonicotinoid that has been shown to harm both *Apis* and non-*Apis* bees [30,37,38,39]. For example, in the neotropical bumble bee *Bombus ephippiatus*, bee survival and colony growth were significantly reduced when exposed to field-realistic levels of imidacloprid [40]. Imidacloprid-induced impairment of mushroom bodies and behavior have also been demonstrated in the native stingless bee *Melipona quadrifasciata anthidioides* [41]. Furthermore, this pesticide was found to be more toxic to the native stingless bee *Nannnotrigona perilampoides* compared to other pesticides, and *N. perilampoides* was more sensitive to imidacloprid than other stingless bee species [33]. An additional importance of *N. perilampoides* is that it is a widespread stingless bee species in Mexico, found both in rural and in urban environments [23,24]; furthermore, it has the potential to be used in commercial pollination [42]. It therefore represents an important model stingless bee species, whose sensitivity to imidacloprid deserves closer scrutiny.

Insects have evolved various detoxification mechanisms to survive natural plant and environmental toxins (xenobiotics), which they also employ in response to insecticides [43]. Insecticide detoxification is one such mechanism; in this case, detoxification is carried out by enzymes that metabolize xenobiotics, including pesticides [44,45]. Though detoxification may allow insects to overcome insecticides, the degree of detoxification differs greatly among insect species, which results in differing toxicity among different stages, populations, and species of insects [43]. Pesticides also have an effect on the immune systems of insects, which include both cellular and humoral responses [46]. The humoral response is mainly elicited by soluble compounds such as antimicrobial peptides (AMPs), which includes apidaecin, hymenoptaecin, abaecin, and defensin [47,48]. Pesticide exposure has been shown to lower global AMP production, further compromising an already frail immune system [49,50,51]. Unfortunately, there is little research on the characterization of detoxification systems and immune responses in stingless bees [52] with which to understand their response and defense against diverse environmental stressors. 

The aim of this study was to study the effect of exposure of the stingless bee *N. perilampoides* to the commonly used neonicotinoid, imidacloprid. To do so, we first calculated the LC_50_ of *N. perilampoides*, then chronically exposed bees for 10 days to two imidacloprid concentrations: LC_10_ and LC_20_. The effects on survival, food consumption, and the abundance of transcripts of immunity and detoxification genes were then quantified to evaluate the sensitivity of the species to the insecticide. 

## 2. Materials and Methods

### 2.1. Nannnotrigona Perilampoides Bees

Six *N. perilampoides* colonies were chosen from the Meliponario at the Faculty of Veterinary Medicine-UADY. To ensure that the experimental bees were not exposed to pesticides, we collected young worker bees from each colony that were identified as being between 1 and 3 days old based on the degree of pigmentation of the cuticle [53]. Eleven bees from each selected colony were placed in one of four plastic containers, for a total of approximately 66 bees per container, with the six experimental colonies represented in each container. The four groups of bees were kept in a climatic chamber at a temperature of 30–32 °C and a humidity of 70–75% until they were 14 days old. Each group received 50% (*w*/*v*) aqueous sugar (sucrose) solution (hereafter: sugar syrup) ad libitum and 1 g of pollen collected from the experimental colonies.

### 2.2. Pesticides

We used analytical grade imidacloprid (Sigma-Aldrich, catalog# 46341—100 µg, St. Louis, CA, USA) and, as a positive control, dimethoate (Sigma-Aldrich, catalogue 59824—5 mg, St. Louis, CA, USA). We dissolved the pesticides in ddH_2_O containing 15% acetone to obtain stock solutions with concentrations of 20 ng/µL for imidacloprid and 40 µg/mL for dimethoate, which were stored at 20 °C to avoid degradation. Aliquots from the original stocks were gradually diluted with 50% sugar syrup until the desired concentration of each pesticide was achieved. 

### 2.3. Determination of LC_50_

The acute oral toxicity (LC_50_) of imidacloprid was calculated using the bees that reached the age of 14 days, when worker bees are typically foragers. We chose forager bees over newly emerged bees because they are more likely to be directly exposed to contaminated nectar [54]. Six serially diluted concentrations of imidacloprid in a geometric series with a factor 2 (0.3, 0.6, 1.2, 2.4, 4.8 and 9.6 ng/µL) were prepared in 50% (*w*/*v*) sugar solution (sugar syrup) and used to determine the LC_50_. Batches of 10 bees at 14 days of age were kept in a glass container (240 mL), giving a total of 24 cages of 10 bees. Bees then received 100 µL of sugar syrup containing the tested concentrations or sugar syrup free of pesticide (control), in Eppendorf tubes (0.2 mL). We also provided each cage with 500 mg of pollen. The bioassays were carried out for 48h under laboratory conditions. Each concentration was performed in triplicate (three cages with 10 bees each).

Dimethoate at a concentration of 15 µg/mL was used as a toxic standard substance (positive reference), and treated identically to those receiving imidacloprid. The experimental groups were maintained in a climatic chamber at 30–32 °C and a humidity of 70–75%. 

Sugar syrup consumption was measured after 24 and 48 h. To accomplish this, each Eppendorf tube was collected and weighed on a daily basis to calculate sugar syrup consumption, after which each cage received a new Eppendorf tube containing freshly prepared sugar syrup spiked with the corresponding treatment. At the same time, the number of dead bees in each cage was recorded. The LC_50_ for imidacloprid was then calculated using the LdP Line program using the log-probit model (Ehabsoft (http://www.ehabsoft.com/ldpline, accessed on 20 February 2020). The control groups had no death after 48 h, whereas the dimethoate group had a mortality rate of 93%. 

### 2.4. Effects of Imidacloprid at LC_10_ and LC_20_ Concentrations

After we calculated the LC_50_ (1.93 ng/µL), we chronically exposed *N. perilampoides* forager bees to imidacloprid at LC_10_ (0.45 ng/µL) and LC_20_ (0.74 ng/µL) for 10 days to test for potential effects on survival, food consumption, and the expression of immunity and detoxification encoding genes. The dosages used in our study were field relevant, correlating with imidacloprid residues (0.2—0.82 ng/g) found in honey from various countries [55,56,57,58], and were 2.7- and 1.3-fold lower than the concentration of imidacloprid detected in honey from central Mexico (6 ng/g) [59]. 

To prepare the bees for the different treatments, we used a similar setup to that described above for determining the LC_50_, using bees obtained from the same six experimental colonies. Dimethoate at a concentration of 5 µg/mL was again used as positive control treatment. We therefore had four treatment groups (Control, Imd (LC_10_), Imd (LC_20_), and dimethoate), with four cages per treatment, though now with 15 bees per cage. 

Similar to the LC_50_ bioassay, bees in each cage received 100 µL of sugar syrup containing either 0.45 ng/µL (LC_10_) or 0.74 ng/µL (LC_20_) of impidacloprid, or sugar syrup free of pesticide (control), for 10 days. We exposed *N. perilampoides* to imidacloprid for 10 days in accordance with the International Commission for Plant Pollinator Relationships (ICPPR) standard 10-day test duration with honey bees [60]. At the start of the experiment, each cage received 500 mg of pollen collected from the experimental colonies. The number of deaths and the amount of sugar syrup consumed per cage, and per individual bee, were recorded daily. At 4 and 8 days after exposure, subsamples of two bees per cage (8 bees per treatment) were collected individually in Eppendorf tubes and stored at −80 °C for quantification of gene expression.

### 2.5. Gene Expression

Total RNA was extracted from the guts of 6 individual bees per treatment at 4 days and 6 individuals at 8 days after exposure. We used an RNA Mini Kit (Quick-Start Protocol-Qiagen, CA, USA) following the manufacturer’s instructions for isolation of total RNA. Genomic DNA contamination was removed from samples by using a DNAse I digestion step (DNA-free kit, Ambion, CA, USA). RNA concentration and purity was measured with a NanoDrop One^®^ (Thermo Scientific NanoDrop Technologies, LLC, Wilmington, DE, USA), and the quality of the RNA was assessed by resolving it by 1.5% agarose gel electrophoresis at 80 V for 30 min. cDNA was synthesized using 0.5 µg of RNA per sample and with a final concentration of 50 Units/µL MultiScribeTM Reverse Transcriptase (Invitrogen/Life Technologies, CA, USA) according to the manufacturer’s recommended protocol. The conditions used for reverse transcription were as follows: 5 min at 25 °C, 10 min at 42 °C, and 15 min at 70 °C.

We used real-time quantitative PCR (RT-qPCR) to quantify the expression of immunity and detoxification-encoding genes in *N. perilampoides* bees in response to LC_10_ and LC_20_ concentrations of imidacloprid (Table 1). We selected three genes with well-documented involvement in insect immune responses, defensin1, hymenopteacin and abaecin; these are part of the Toll/Antimicrobial peptide, or the Imd pathways [61,62]. We also selected one gene (glutathione S transferase D1: GSTD1) as a representative of antioxidant enzyme families that are known to target pesticides and secondary metabolites as part of a detoxification response in honey bees [63,64]. The primers for these genes were obtained from earlier honey bee studies [51,64]. These genes have been used for comparable studies with honey bees [51,63,65,66].

RT-qPCR was performed in a thermocycler CFX-96 Real-Time PCR System (Bio-Rad, Hercules, CA, USA). Amplification of cDNA was performed using SsoAdvanced Universal Inhibitor-Tolerant SYBR^®^ Green Supermix for detection of a signal (Bio-Rad). Each RT-qPCR reaction mix contained 2 μL of cDNA, 7 μL of SYBR^®^ Green Supermix, 1 μL of each gene-specific primer (0.4 μM), and 1 μL of PCR-grade water. Three technical replicates were run per sample in a 96-well PCR plate. Amplification runs were initiated at 95 °C for 30 s, followed by 39 cycles of 95 °C for 15 s, annealing at 59 °C for 30 s, and extension at 72 °C for 30 s. The specificity and accuracy of RT-qPCR products were validated for all samples by examining the melt curve at the end of the program to ensure that only one product of the correct melt temperature (as expected for *A. mellifera*) was amplified in each reaction. Furthermore, primers matched well the homologous sequences of available stingless bee genomes (*Heterotrigona itama* and *Melipona quadrifasciata*), suggesting that we had amplified the homolog of the honey bee genes in *N. perilampoides*.

The relative quantification of the target genes was normalized using RPS5 as an endogenous reference control, which was chosen after Bio-Rad CFX Maestro software confirmed its stability. The efficiency of each set of primers was determined by the use of a standard curve of serial dilutions of cDNA. Reaction conditions were optimized so that the coefficient of determination (R^2^) was at least 0.99 and efficiencies were >91%, where efficiency = 10^(−1/slope of standard curve)^. Relative expression of the genes investigated was calculated using the comparative CT (ΔΔCT) method [67]. Gene expression analysis was carried out using six biological replicates per treatment and three technical replicates for each biological replicate.

### 2.6. Statistical Analysis

Survival analysis was performed with the R package coxme [68] in R using mixed-effects Cox proportional hazard models, with ‘cage’ as a nested random effect; models with ‘cage’ gave a better model fit (lower AIC value) than models without this random effect so cage was retained in the final model. Right censored samples (bees removed at days four and eight for analysis of gene expression) were recorded in the dataset and incorporated in the Cox proportional hazard models. To test for differences between treatments, we performed linear contrasts (Tukey test) of Cox proportional hazard coefficients (hazard ratios) using the R package multcomp [69].

To test for treatment effects on daily sugar syrup consumption, we used one way analysis of variance (ANOVA) followed by Tukey’s post hoc test. To compare the change in abundances of transcripts of genes studied in response to tested concentrations of imidacloprid at 4 and 8 days of exposure, normality of the relative expression was tested using the Shapiro–Wilk normality test, then we used ANOVA (Type III) tests in a generalized linear model (GLM). Treatment and time of assessment were used as independent, fixed factors (predictors). To test for significant interactive effects of pesticide treatment and time of assessment, we inspected the treatment × time interaction terms in all models, followed by a pairwise Wilcoxon rank sum exact test with Bonferroni correction. A significance level of 0.05 was used to define a test’s significance. GraphPad Prism 8.00 for Windows was used to visualize the data (www.graphpad.com, accessed on 10 February 2022).

## 3. Results

### 3.1. Acute Oral Toxicity of Imidacloprid (LC_50_)

The oral lethal concentrations (ng µL^−1^) of imidacloprid required to kill 10, 20, and 50% (LC_5_, LC_20,_ and LC_50_) of *N. perilampoides* foragers after 48 h of acute exposure are reported (Table 2). The calculated LC_50_ value of imidacloprid to *N. perilampoides* foragers was 1.93 ng/µL.

### 3.2. Effects of Imidacloprid at LC_10_ and LC_20_ Concentrations

#### 3.2.1. Effects on Survival and Food Consumption

**Figure 1:**
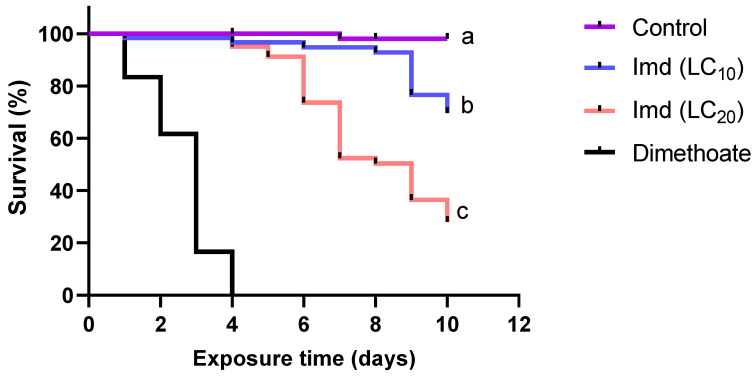
Kaplan–Meier survival curves of the effect of imidacloprid (Imd) and dimethoate (reference toxic chemical) insecticides on *Nannotrigona perilampoides* stingless bees. Bees (*n* = 15 bees per cage, *n* = 4 cages per treatment) were fed with Imd LC_10_ or LC_20_, or a control solution for 10 days. Different lowercase letters indicate the significant differences between the respective sublethal concentrations of Imd and control on survival of bees (*n* = 240; Cox proportional hazard, *p* < 0.05). For statistical details, see Table 3.

When we exposed *N. perilampoides* foragers in the laboratory to sugar syrup spiked with imidacloprid for 10 days to either LC_10_ or LC_20_, survival was significantly reduced (−30% in LC_10_ and −72% in LC_20_ treated bees) (Cox proportional hazard model, *p* < 0.01) compared to non-exposed control bees (Figure 1). Survival of bees that were chronically exposed to LC_20_ of imidacloprid was markedly (and significantly) reduced compared to bees exposed to LC_10_ (*p* < 0.0001) (Figure 1). Model-averaged β coefficients (standardized effect size of the hazard) revealed that the hazard ratio (HR) of imidacloprid LC_20_ is four times greater than that of imidacloprid LC_10_ (HR) (Table 3), with bees exposed to imidacloprid LC_20_ surviving only a median of 9 days. 

**Figure 2:**
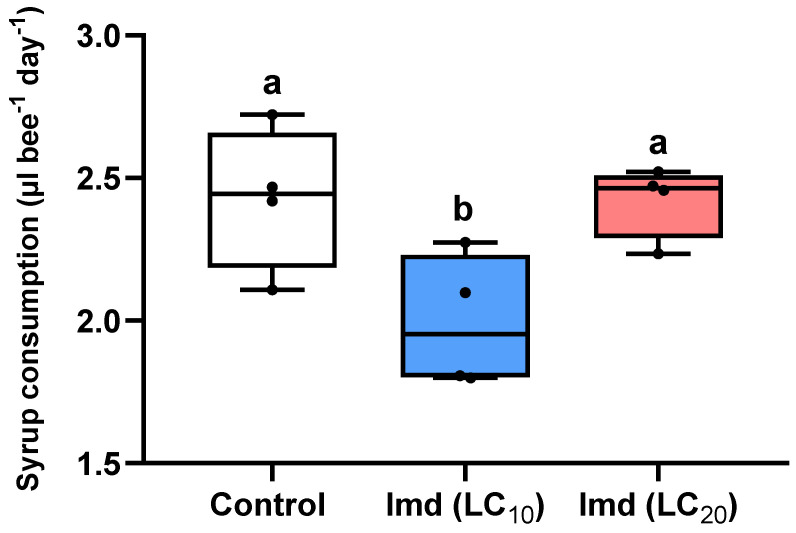
Effects of exposure to LC_10_ or LC_20_ of the insecticide imidacloprid (Imd) on average daily syrup consumption (reported in µL/bee/day, mean ± Standard Error of the Mean, SEM) by *Nannotrigona perilampoides* stingless bees. Bees (*n* = 15 bees per cage, *n* = 4 cages per treatment) were fed with Imd (LC_10_ or LC_20_), or a control solution for 10 days. Symbols on the box plot represent maximum and minimum values (whiskers: ┬ ┴) and mean values (-). Different lowercase letters indicate significant differences between the respective Imd concentration and control on food consumption (ANOVA, *p* < 0.05).

Chronic exposure to imidacloprid at an LC_10_ concentration significantly reduced bees’ daily sugar syrup consumption, compared to bees fed pesticide-free sugar syrup (control), and bees fed sugar syrup containing the LC_20_ of imidacloprid (ANOVA, F = 5.52, df = 2, *p* = 0.02) (Figure 2).

#### 3.2.2. Effects on Gene Expression

**Figure 3:**
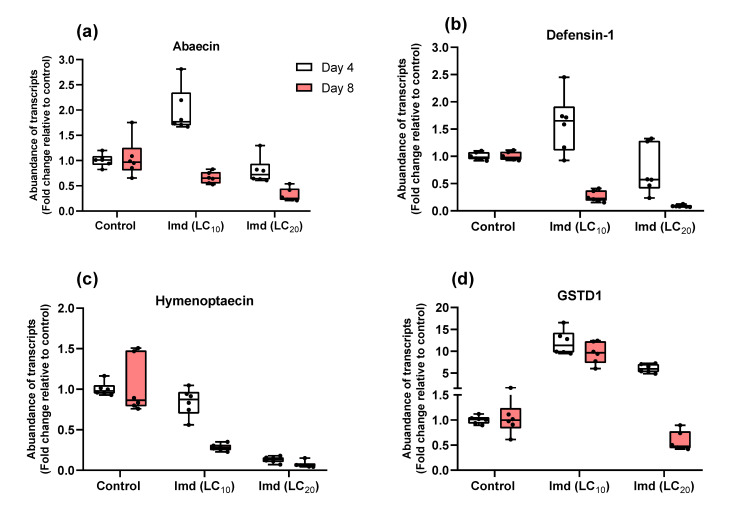
Fold-change in the abundance of transcripts of innate immune (**a**–**c**) and detoxification-related (**d**) genes in adult *Nannotrigona perilampoides* stingless bees. Bees (*n* = 15 bees per cage, *n* = 4 cages per treatment) were fed with sugar syrup spiked with Imd (LC_10_ or LC_20_), or a control solution for 10 days. Symbols on the box plots show the minimum and maximum values (*n* = 6 bees) (whiskers: ┬ ┴) and mean values (-), with jittered data points. For statistical details, see Table 4.

When we compared gene expression of the four genes studied (abaecin, defensin1, hymenopteacin, and GSTD1) in bees fed sugar syrup containing either an LC_10_ or LC_20_ concentration of imidacloprid or the control at 4 and 8 days of exposure, we found significant treatment x time interaction terms for all genes investigated (*p* < 0.001), indicating that the effect of the imidacloprid exposure on gene expression differed over time (see Figure 3 and Table 4 for more details). Results of pairwise comparisons using Wilcoxon rank sum exact tests revealed several significantly different treatment pairs (Table 4). For example, in bees exposed to LC_10_ imidacloprid, only the GSTD1 gene was significantly modulated compared to the control at day four, whereas at day eight the expression of all genes except abaecin changed significantly, indicating that the duration or repeated exposure to imidacloprid, even at a low concentration, has an effect on gene expression (Table 4).

Gene expression was always relatively low in bees exposed to imidacloprid LC_10_ or LC_20_ after 8 days compared to after 4 days. Interestingly, the GSTD1 gene expression in bees exposed to LC_20_ changed over time, with it being upregulated on day four and downregulated on day eight, whereas it was upregulated on both days four and eight in bees exposed to LC_10_ (Figure 3), demonstrating the effect of repeated exposure to a slightly higher concentration of imidacloprid, likely reducing the bees’ detoxification ability.

## 4. Discussion

The current concern about bee population losses and their link to neonicotinoid use has focused research attention on honey bees [70]. However, because native pollinators also play an important role in pollination, it is crucial to include them in pesticide risk assessments. We found that exposing native *N. perilampoides* stingless bees to imidacloprid markedly reduced bee survival and food consumption, while also modulating the expression of detoxification and immune-related genes, suggesting that this and other stingless bee species may be far more sensitive than honey bees to imidacloprid.

The LC_50_ value of imidacloprid in *N. perilampoides* stingless bee foragers determined in our study was 1.9 ng/µL. Given that bees consume an average of 2.4 microliters (µL) of sugar syrup per day, as calculated in the current study, we calculate and predict the LD_50_ by multiplying the LC_50_ value by the 4.8 µL of imidacloprid–sucrose solution consumed in 48 h by each forager bee. The estimated oral LD_50_ was 9.12 ng/bee, which is almost an order of magnitude lower than the LD_50_ of imidacloprid in honey bees (41–81 ng/bee) [71,72] and bumble bees (38 ng/bee) [73], indicating this species’ high sensitivity to imidacloprid. This could be due to the bee’s small size, as small bees are more sensitive than large ones due to the high surface/volume ratio [74,75]; indeed, workers and males of *N. perilampoides* are small, measuring about 4 mm in body length [76]. Our results are in agreement with others, who found that imidacloprid was particularly toxic to *N. perilampoides* compared with other pesticides. In support of our conjecture that honey bees may be poor proxies for the sensitivity of stingless bees to imidacloprid, [77] have recently found four Brazilian stingless bee species to also show much greater sensitivity to this insecticide than honey bees. 

Typical risk assessments only address a pesticide’s acute toxicity after topical or oral exposure for 24 or 48 h, overlooking the deleterious consequences of long-term exposure to pesticide residues [78]. In the current study, we exposed *N. perilampoides* stingless bees to imidacloprid insecticide at LC_10_ and LC_20_ for 10 days and found a substantial decrease in bee survival, particularly in bees exposed to imidacloprid LC_20_ (0.74 ng/µL). Previous research has found that chronic exposure to imidacloprid concentrations less than 20 ng/µL had only a minor impact on honey bee survival under laboratory and semi-field in-hive experiments [79,80,81]; clearly, *N. perilampoides* is far more sensitive to imidacloprid than the honey bee. Furthermore, we found that *N. perilampoides* bees chronically exposed to imidacloprid LC_10_ consumed less sugar syrup than control and imidacloprid LC_20_ bees. As a result, we calculated the cumulative dose over a 10-day chronic exposure and found that *N. perilampoides* bees exposed to LC_10_ had a cumulative dose of 8 ng/bee (98.4% of calculated LD_50_ of 9.12 ng/bee), whereas bees exposed to LC_20_ had a cumulative dose of 16 ng/bee (196.4% of the LD_50_). This could explain why the hazard ratio (HR) of imidacloprid LC_20_ is four times higher than the hazard ratio (HR) of imidacloprid LC_10_. 

We expected feeding avoidance to occur at a high insecticide concentration, for example our LC_20_ treatment, but not at our LC_10_ treatment; however, we saw the opposite. Our result could be explained by bees exposed to LC_10_ attempting to consume as little sugar syrup as possible as a behavioral defense mechanism to avoid imidacloprid intoxication. In contrast, bees repeatedly exposed to imidacloprid at a two-fold higher concentration (LC_20_) may have experienced physiological stress; sugar syrup consumption may then have been necessary for them to meet energy requirements for metabolic pathways and detoxifying capabilities [81]. Our findings nevertheless differ from previous research that found no significant differences in daily syrup consumption of honey bees exposed to imidacloprid concentrations less than 20 ng/mL [50], or that found that bees even preferred and consumed more food containing neonicotinoid pesticides [82,83]. The toxicity and sensitivity of different stages, populations, and species of insects may be related to the type of pesticide, mode of action, duration of exposure, pesticide dose or concentration, and timing of exposure [43,51,84]. As a result, understanding how native stingless bees respond to and defend against agrochemicals is crucial in allowing recommendations to be formulated for their use in the tropics.

Pesticides have also been shown to weaken bee immune systems and decrease their detoxification capacity [46,47,48]. We found that repeated exposure to imidacloprid at lesser concentrations altered the expression of immune-related genes (abaecin, defensin1, and hymenopteacin); moreover, significant treatment x time interaction terms were observed, showing that gene expression varied over time. These findings are not consistent with the reports of previous studies that abaecin expression in *A. mellifera* adults was unaffected by exposure to coumaphos, tau-fluvalinate, imidacloprid, and spinosad [85,86,87] as well as in *M. quadrifasciata* workers exposed to azadirachtin or spinosad insecticides [52]. However, similar to our findings, a significant increase in the expression of AMP genes abaecin, apidaecin, and hymenoptaecin, was observed in bumble bees exposed to moderate to high concentrations of imidacloprid, and responses were time and dose dependent [88]. Pesticide exposure has been shown to lower global AMP production, further compromising an already frail immune system [48,49,50]. Given that gene expression was always lower in bees exposed to imidacloprid LC_10_ or LC_20_ after 8 days than it was after 4 days, lower AMP production is expected, and as a result, the immune system of the bees may be compromised, making them more susceptible to pathogens [89].

Pesticides and secondary metabolites are known to be targeted by antioxidant enzyme families as part of a detoxification response in honey bees [48,63]. The enzyme encoded by GSTD1, as an antioxidant member, is thought to play a function in honey bee (*Apis mellifera*) oxidative stress tolerance [90]. Previous research found an increase in GSTDI expression in honey bees in response to low concentrations of imidacloprid exposure in both laboratory and field conditions [48,91]. In the current study, we found that the expression of GSTD1 in *N. perilampoides* changed over time in bees exposed to LC_20_, with this gene being upregulated on day four and downregulated on day eight, although not significantly different from control; in contrast, it was upregulated on both days four and eight in bees exposed to LC_10_. The reason for these differences in gene expression may be that cumulative toxicity was still minimal in bees treated with imidacloprid LC_10_ or LC_20_ at day four of exposure; an increase in GSTD1 expression at day four could be explained as a detoxifying strategy. This strategy may have been inhibited at day eight in bees exposed to LC_20_ due to increased cumulative toxicity (1.5-fold greater than LD_50_). This could also explain why *N. perilampoides* bees exposed to only LC_10_ after 8 days could still activate detoxification mechanisms. More research is needed, however, to characterize other detoxification-encoding genes that are part of the cytochrome P450 pathway in order to gain a better insight into how stingless bees, and *N. perilampoides* in particular, respond to pesticide toxicity.

## 5. Conclusions

Here we found for the first time that long-term exposure of *N. perilampoides* stingless bees to imidacloprid insecticide at LC_10_ and LC_20_ concentrations reduced bee survival and food consumption, while they also modulated the expression of detoxification and immune-related genes; effects were time and dose dependent. Our data indicate that *N. perilampoides* stingless bees are very sensitive to the effects of imidacloprid, even at low concentrations. It is critical to act to protect this important bee species for the ecology and agriculture of Mexico and Latin America.

## Figures and Tables

**Table 1:** Sequences of primers used in the study.

Gene Description	Category	F. Primer	Length (pb)	Eff.
Ribosomal protein S5a (RPS5)	Reference (housekeeping)	F-AATTATTTGGTCGCTGGAATTG	115	1.98
		R-TAACGTCCAGCAGAATGTGGTA		
Glutathione S-transferase D1 (GSTD1)	Detoxification	F-CTTGCCGATTTAAGCATCGT	142	1.91
		R-ACCCAGCGTTGTTGTACTCC		
Abaecin	Immunity	F-CAGCATTCGCATACGTACCA	72	1.95
		R-GACCAGGAAACGTTGGAAAC		
Defensin 1	Immunity	F-TGCGCTGCTAACTGTCTCAG	119	1.94
		R-AATGGCACTTAACCGAAACG		
Hymenoptaecin	Immunity	F-CTCTTCTGTGCCGTTGCATA	200	1.92
		R-GCGTCTCCTGTCATTCCATT		

**Table 2:** The oral lethal concentrations (ng µL^−1^) of imidacloprid required to kill 10, 20, and 50% (LC_10_, LC_20_, and LC_50_) of *N. perilampoides* foragers after 48 h acute exposure.

Pesticide	*n*	LC_10_ (95% CI) (ng. µL^−1^)	LC_20_ (95% CI) (ng. µL^−1^)	LC_50_ (95% CI (ng. µL^−1^)	Slope (Mean ± SE)
Imidacloprid	180	0.45 (0.25–0.65)	0.74 (0.49–0.99)	1.93 (1.49–2.51)	2.03 ± 0.26

*n*, number of bees tested; CI, confidence interval; SE, standard error mean.

**Table 3:** Impact of exposure to imidacloprid (LC_10_ or LC_20_) insecticide on *Nannotrigona perilampoides* stingless bees’ survival based on Cox proportional hazard models; model-averaged ß coefficients (standardized effect size of the hazard, where higher β indicates a higher risk of death) of the two concentrations of imidacloprid (LC_10_ or LC_20_) insecticide and the exp. ß, equivalent to the hazard ratio obtained from a Cox proportional hazard model in comparison to control. In bold are treatment effects that were significantly different from control by post hoc Tukey tests (with Bonferroni correction for multiple comparisons).

Treatment	ß	SE of ß Coefficient (+/−)	exp. ß	Z	*p*
Imd (LC10)	2.77	1.03	15.97	2.68	**0.02**
Imd (LC20)	4.20	1.01	67.02	4.13	**<0.001**

**Table 4:** Pairwise comparisons using Wilcoxon rank sum exact test with Bonferroni correction for the effects of exposure to imidacloprid insecticide at LC_10_ or LC_20_ on the expression of some detoxification and immunity-related genes in *Nannotrigona perilampoides* stingless bees after 4 and 8 days of exposure.

*p*-Value				
Treatment × Time	Abaecin	Defensin-1	Hymenoptaecin	GSTD1
**LC10_4 vs. Cont_4**	0.07	0.61	0.97	0.03
**LC20_4 vs. Cont_4**	0.97	1.00	0.03	0.03
**LC10_8 vs. Cont_8**	0.22	0.03	0.03	0.03
**LC20_8 vs. Cont_8**	0.03	0.03	0.03	0.13
**LC10_4 vs. LC10_8**	0.03	0.03	0.03	0.21
**LC20_4 vs. LC20_8**	0.03	0.03	0.39	0.03
**LC10_4 vs. LC20_4**	0.03	0.39	0.03	0.03
**LC10_8 vs. LC20_8**	0.06	0.03	0.03	0.03

## Data Availability

The data presented in this study are available on request from the corresponding author.
